# Spatial variation of overweight/obesity and associated factor among reproductive age group women in Ethiopia, evidence from EDHS 2016

**DOI:** 10.1371/journal.pone.0277955

**Published:** 2022-11-29

**Authors:** Ermias Bekele Enyew, Abraham Yeneneh Birhanu, Wondwossen Zemene Mewosha

**Affiliations:** 1 Department of Health Informatics, Mettu University, Mettu, Ethiopia; 2 Department of Health Informatics, College of Health Medicine and Health Science, Gondar University, Gondar, Ethiopia; University of Uyo, NIGERIA

## Abstract

**Background:**

Globally, at least 4.7 million people die from being overweight or obese. In Ethiopia, the level of overweight and obesity among women grew from 3% to 8%. However, as far as my literature searching, studies concerning the spatial variation of overweight/obesity and factors associated are not researched in Ethiopia using geospatial techniques. Therefore, this study aimed to explore the spatial variation of overweight/obesity and factor associated among reproductive age group women in Ethiopia using geospatial techniques.

**Mothed:**

A total weighted sample of 10,928 reproductive age women were included in the study. ArcGIS version10.7 was used to explore the spatial variation of overweight/obesity. Bernoulli based model was used to analyze the purely spatial cluster detection of overweight/obesity through SaTScan version 9.6.1 software. Ordinary Least Square analysis and geographically weighted regression analysis was employed to assess the association between an outcome variable and explanatory variables by using ArcGIS 10.7 software. P value of less than 0.05 was used to declare statically significant.

**Result:**

The spatial distribution of overweight/obesity in Ethiopia was clustered. Statistically, a significant-high hot spot overweight/obesity was identified at Addis Ababa, harrari, Dire Dawa. SaTScan identified 66 primary spatial clusters (RR = 4.17, P < 0.001) located at Addis Ababa, southeast amhara, central part of oromia region and northern part of SNNP region. In geographically weighted regression, rich wealth index, women’s age (35–39 and 40–44 years), watching TV, internet use and not working were statistically significant that affecting spatial variation of overweight/obesity.

**Conclusion:**

In Ethiopia, overweight/obesity varies across the region. Statistically, significant-high hot spots of overweight/obesity were detected in Addis Ababa, Harari, Dire Dawa, some parts of Amhara and afar region, most of the Oromia and Somalia region, and the South Nation Nationality and People region of Ethiopia. Therefore, the ministry of health and the Ethiopian public health institute, try to initiate policies and practices that could include providing funding for physical education as well as recreational centers in communities most in need. In addition, public and private mass media create awareness of healthy lifestyles is promoted by health education regarding increased physical activity and reduced sedentary behavior through various media platforms.

## Background

Overweight and obesity are as a result of energy imbalance that consumes more calories than what is equivalently expended in physical activities [1]. The World Health Organization (WHO) has announced that overweight and obesity are the fifth risk factor for global death [2]. In 2017, at least 4.7 million people die from being overweight or obese globally each year, and approximately 1.9 billion adults, accounting 40% of women were overweight and 15% women were obese. Additionally, 44% of diabetes, 3% of ischemic heart diseases, and 7%–41% of certain cancers are caused by overweight or obesity [1, 3].

In sub Saharan Africa, the high prevalence of overweight and obesity among reproductive age group women was shown in Uganda (54.4%), in Nigeria (64.1%), in Tanzania (82.7) and in South Africa (70.5%) [4]. In Ethiopia, the level of overweight and obesity among women grew from 3% in 2000 to 8% in 2016 [5]. Similarly, numerous pocket area studies among women stated that the prevalence of overweight or obesity was observed in Addis Ababa with (20.6%), Hawassa (36.4%), Dessie (26.7%) and the southern region (56.2%) [6–9].

Prior studies tried to identify overweight/obesity were associated with many factors including excessive consumption of alcohol drinking, older age, high wealth index, eating snack, access to improved water, higher education, not working, use of hormonal contraception, urban residence and sedentary lifestyle habits [8, 10, 11]. Overweight and obesity in women increases to exposes various health issues, including miscarriage, perinatal mortality, fetal macrosomia, congenital malformations, instrumental delivery, preterm delivery, pre-eclampsia, postpartum hemorrhage, high birth weight baby and infant overweight [12–14].

Previous studies have investigated the prevalence of and associated factor overweight and obesity among women of reproductive age. However, as far as my literature searching, studies concerning spatial variation of overweight/obesity and factors associated are not researched in Ethiopia using geospatial techniques. This first geographically weighted analysis on overweight/obesity and geographically vary risk factors among reproductive age women in Ethiopia have been provided. Therefore, the aim of this study was to assess the spatial variation of overweight/obesity and factors associated among women in Ethiopia important to get information which help to take geographic based interventions and which contributes the prevention and control of these emerging public challenges in Ethiopia.

## Methods and material

### Study design, period and area

**Fig 1:**
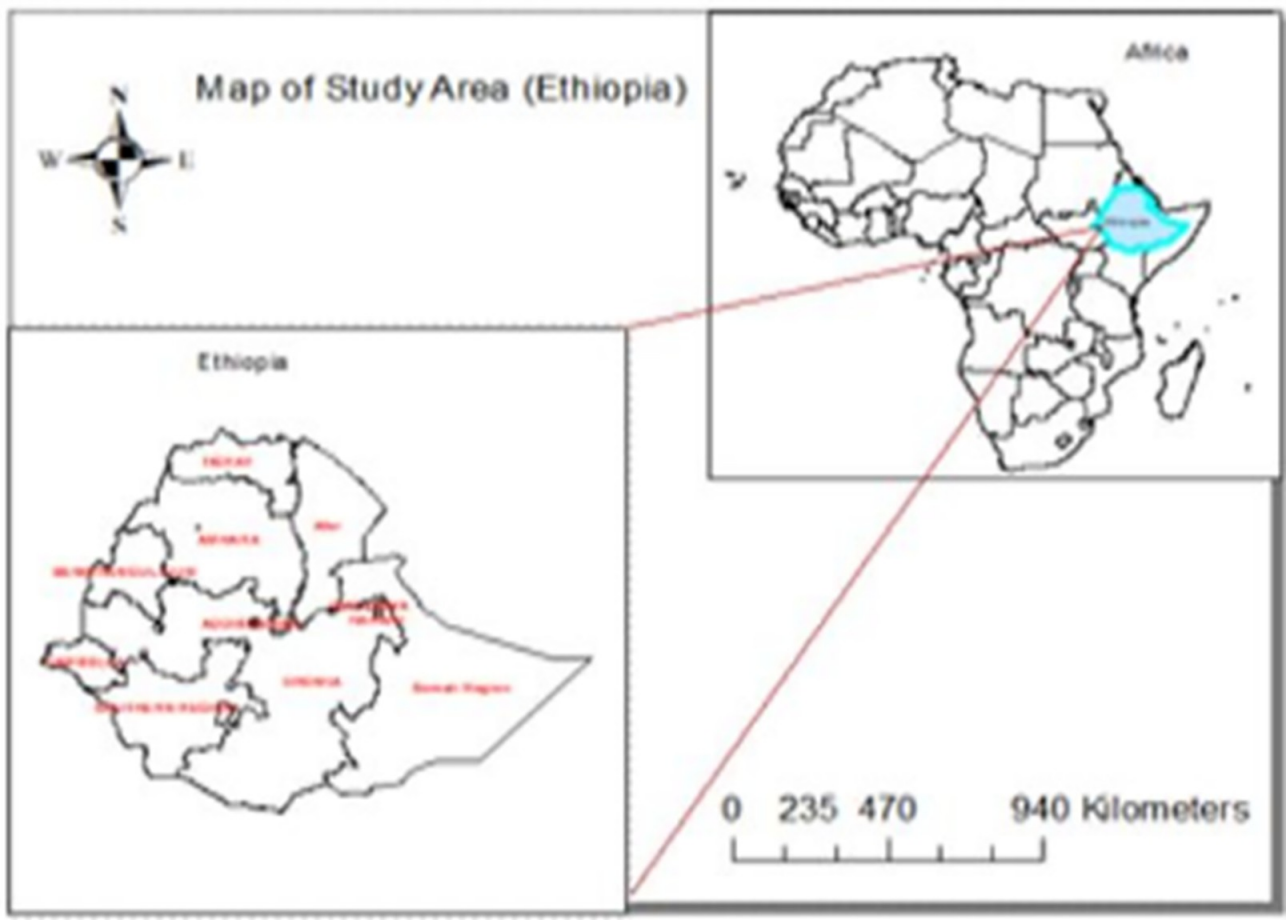
Map of study area (Ethiopia) Shape file source: CSA, 2013; URL: https://africaopendata.org/dataset/ethiopia-shapefiles.

In EDHS 2016, a community-based cross-sectional study was conducted by the Central Statistical Agency (CSA) from January 18 to June 27, 2016, in Ethiopia [15]. It has nine Regional states (Afar, Amhara, Benishangul-Gumuz, Gambella, Harari, Oromia, Somali, Southern Nations, Nationalities, and People’s Region (SNNP) and Tigray) and two city Administrative (Addis Ababa and Dire-Dawa) (Fig 1).

### Source and study population

The source of population was all reproductive-age women within five years before the survey in Ethiopia. A weighted total of 10,928 reproductive age group women who had a complete answer to all variables of interest were included from the total 15,683 women aged 15–49 years were interviewed. All women those who had underweight and women for whom there was missing information on height and/or weight and/or women those who had pregnant and observation from enumeration areas with zero coordinate were excluded. The data was acquired from the Demographic and Health Surveys (DHS) Program by requesting for this work and accessed www.dhsprogram.com website.

### Data collection tool and procedures

In the 2016 EDHS, a standardized and validated questionnaire were adapted from the DHS Program’s standard questionnaires in a way to reveal the population and health issues relevant to Ethiopia. A two stage stratified sampling technique was employed to select representative samples for the country as whole. The regions of the country were stratified into urban and rural areas. Then, samples of enumeration areas (EAs) were selected in each stratum in two stages. In the first stage, 645 EAs were selected with probability proportional to the EA size. The EA size is the number of residential households in the EA as determined in the 2007 Ethiopian Population and Housing Census. In the second stage, a fixed number of 28 households per cluster were selected randomly from the household listing [15]. For the purpose of this study, the women’s data (IR) from the 2016 EDHS was utilized. The detailed sampling procedure is available in the Ethiopian Demographic and Health Survey reports from Measure DHS website (www.dhsprogram.com).

### Study variables

#### Outcome variable

The outcome variable of this study was Body Mass Index (BMI). BMI is a measure for quantifying nutritional status in adults which is calculated by using the formula weight in kg divided by height in meter square. This study utilized the WHO-specific cut-offs value to categorize BMI of the reproductive age group women. Normal weight: 18.5–24.9 kg/m2, Overweight: 25.0–29.99 kg/m2 and Obese: ≥30.0 kg/m2 [1]. For the purpose of this study, a BMI ≥25.0 kg/m2 was categorized as overweight/obesity and coded as 1, while a BMI 18.5–24.99 kg/m2 was categorized as normal and coded as 0.

#### Independent variable

**Socio-demographic factor** marital status (labelled as single, married, divorced/separated and widowed), Women’s education status (labelled as no education, primary, secondary, and higher education, maternal occupation (labelled as working or not working), parity(labelled as 0 children, 1–3 children and 4 and above children), wealth index (labelled as poor, middle and rich) and maternal age.

**Behavioral factors** such as alcohol use (labeled as yes and No), cigarette smoking (labeled as yes and No) and contraceptive use (labeled as yes and No).

#### Media exposure

Exposure to mass media (whether the woman read any newspapers/magazines, listened to the radio, or watched television and internet use) were labeled as (less than once a week and at least once a week) and not at all).

### Data management and statistical analysis

#### Spatial autocorrelation analysis and hotspot analysis

The data was clean by STATA version 14.1 software and Microsoft Excel, for data analysis we used Arc GIS 10.7 and SaTScan 9.6. Spatial autocorrelation (Global Moran’s I) statistic measure is used to assess whether overweight/obesity is dispersed, clustered, or randomly distributed in Ethiopia. Moran’s I value close to − 1 shows dispersed overweight/obesity, close to + 1 shows clustered, and if Moran’s I value zero shows randomly distributed and a statistically significant Moran’s I (p < 0.05) leads to rejection of the null hypothesis [16]. Hot Spot Analysis (the Getis-Ord Gi * statistic) of the z-scores and significant p-values tells the features with either hot spot or cold spot values for the clusters spatially [17].

#### Incremental autocorrelation analysis

For measuring spatial autocorrelation for a set of distances, a line graph of those distances and their related Z-scores was generated. The level of spatial clustering and statistical significance are represented by Z-scores. Peak Z -scores show distances where spatial processes promoting clustering are most pronounced. These peak distances are often proper values to use for tools with a Distance Band or Distance Radius parameter. This tool can help to select an appropriate distance threshold or radius for tools that have these parameters, such as hot spot analysis [18].

#### Spatial interpolation

The spatial interpolation technique is used to predict overweight/obesity for unsampled areas based on sampled clusters [19]. We used deterministic and geostatistical interpolation methods. To compare the above interpolation method we employed geostatistical analysis based on result with lowest mean predicted error (MPE) and root mean square predicted error (RMSPE) was the best fitted interpolation technique for overweight/obesity. Those small values indicate that predicted values are close to the observed values and vice versa [20]. For this study ordinary kriging interpolation method was selected, since lowest mean predicted error (MPE) and root mean square predicted error (RMSPE).

#### Spatial scan statistics

Bernoulli based model spatial Kuldorff’s Scan statistics was used to determine the geographical locations of statistically significant spatial window for overweight/obesity using SaTScan version 9.6.1 software [21]. The outcome variable has a Bernoulli distribution, so Bernoulli model was used by applying the Kuldorff’s method for purely spatial analysis. The scanning window that moves across the study area in which women give overweight/obesity was taken as case and those women who give normal body weight was taken as control to fit the Bernoulli model. The default maximum spatial cluster size of < 50% of the population was used as an upper limit and most likely clusters was identified by using p-values and likelihood ratio tests based on 999 Monte Carlo replications. To generate secondary clusters, we employed non-overlapping options by SaTScan version 9.6.1 and ArcGIS software version 10.7 was used to map the cluster and attribute of overweight produced by SaTScan™.

#### Spatial regression

Exploratory Regression was used to find a model that meets the OLS method’s assumptions, all while identifying models with a high Adjusted R2 value. Ordinary Least Square regression (OLS) model is a global model that estimates only one single coefficient per explanatory variable over the entire study region. We used to check assumptions of spatial regression using explanatory regression with the particular tests. The Jarque-Bera test was used to assess the normality assumption for residuals. As residuals are not spatially auto-correlated, the statistically significant Koenker (BP) statistic shows that the relationships modeled are not consistent (either due to non-stationarity or heteroscedasticity). Multicollinearity (Variance Inflation Factor) was used to check redundancy among predictor variables, coefficients have the expected sign and statically significant, and strong adjusted R2 values.

**A geographically weighted regression model** is an extension of the OLS regression model. It gives local parameter estimates to reflect variations over space in the association between an outcome and predictor variables [22]. For geographically weighted regression analysis, the aggregated proportion of overweight/obesity among reproductive-age women and all the predictor variables are considered for each cluster. To determine the predictor variables for overweight/obesity among reproductive-age women, we used a geographically weighted regression model. The model structure of geographically weighted regression written as:

$$Yi=\beta_{0}\;(ui,\;vi)+\sum k\;\beta k\;(ui,\;vi)\;Xik+I$$

Where Yi is the outcome variable, (ui, vi) represents the coordinates of the ith point in space, β0 is the intercept at the (ui, vi) coordinate, βk is the coefficient of the covariate X at the (ui, vi) coordinate, and i is the random error term.

Geographical heterogeneity for each coefficient can be measured by comparing the AICc between the GWR model and the global OLS regression model. The corrected Akaike Information Criteria (AIC) and Adjusted R-squared was used for model comparison of OLS (global model) and GWR (local) model. A model with the lowest AICc value and a higher adjusted R-squared value was used to determine the best fit model for local parameter estimates.

#### Ethics approval and consent to participate

Ethical clearance was obtained from the ethical review board of the University Of Gondar Institute of Public Health, CMHS. The guidelines expressed in Ethiopia’s Declaration of Central Statistical Agency (DCSA) guided the countrywide survey. The survey was also approved by CSA’s Ethical Review Board (ERB), and everyone who decided to take part in it completed a consent form. Permission for data access was acquired from the measure demographic and health survey through an online request by written letter of objective and significance of the study from http://www.dhsprogram.com. Moreover, for Geographic information system coordinates, the coordinates are only for the enumeration area (EA) as a whole and the measured coordinates were randomly displaced within a large geographic area so that no particular enumeration areas can be identified.

## Result

### Sociodemographic characteristics and distribution of overweight/obesity among reproductive age group women in Ethiopia, EDHS 2016

A weighted total of 10,928 participants were included in the study. Out of the 10,928 women, 1,048(9.60%) were overweight/obesity. From the study participant more than 2,226 (20.37%) women were the age between 15–19 years with a mean± SD age of 28.4 (± **0.8**) years. Majority of the participants, 7,028 (64.32%) were married and 8257 (74.56%) were rural dwellers (Table 1).

**Table 1:** Socio-demographic characteristics and distribution of overweight/obesity among reproductive age group women in Ethiopia (n = 10928), EDHS 2016.

			Overweight/obesity	
Characteristics	Weighted frequency(n)	Weighted percentage (%)	No 9,880(90.40%)	Yes 1,048(9.60%)
**Maternal age in year**				
15–19	2,226	20.37	2,122(95.32%)	104(4.68%)
20–24	1,877	17.18	1,756(93.59%)	120(6.41%)
25–29	2,115	19.35	1,916(90.62%)	198(9.38%)
30–34	1,680	15.37	1,455(86.62%)	224(13.38%)
35–39	1,354	12.39	1,185(87.54%)	168(12.46%)
40–44	936	8.57	799(85.38%)	136(14.62%)
45–49	738	6.76	643(87.09%)	95(12.91%)
**Place of residence**				
Urban	2,671	24.44	2,002(74.96%)	668(25.04%)
Rural	8,257	74.56	7,877(95.40%)	379(4.60%)
**Marital status**				
Single	2,847	26.06	2,634(92.52%)	212(7.48%)
Married	7,028	64.32	6,348(90.32%)	680(9.68%)
Widowed	322	2.95	260(80.71%)	62(19.29%)
Divorced	729	6.67	635(87.22%)	93(12.78%)
**Wealth index**				
Poor	3,563	32.61	3,435(96.39%)	128(3.61%)
Middle	2,034	18.62	1,967(96.71%)	66(3.29%)
Rich	5,330	48.77	4,476(83.99%)	853(16.01%)
**Maternal education status**				
No education	5,156	47.19	4,851(94.07%)	305(5.93%)
Primary education	3,771	34.51	3,416(90.57%)	355(9.43%)
Secondary education	1,365	12.49	1,153(84.44%)	212(15.56%)
Higher	634	5.81	459(72.41%)	175(27.59%)
**Maternal working status**				
Not working	7,092	64.90	6,288(88.66%)	804(11.34%)
Working	3,836	35.10	3.518(91.7%)	318(8.3%)
**Parity**				
0 children	3,494	31.97	3,207(91.80%)	286(8.20%)
1–3 children	3,458	31.65	3,009(87.00%)	449(13.00%)
>4 children	3,975	36.38	3,662(92.14%)	312(7.86%)
**frequency of watching television**				
Not at all	7,675	70.23	7,295(95.05%)	379(4.95%)
Less than and a least once a week	3,253	29.77	2,584(79.43%)	669(20.57%)
**frequency of reading/newspaper or magazine**				
Not at all	9,392	85.94	8,669(92.30%)	722(7.70%)
Less than and a least once a week	1,536	14.06	1,210(78.78%)	326(21.22%)
**frequency of listening to radio**				
Not at all	7,183	65.73	6,661(92.72%)	523(7.28%)
Less than and a least once a week	3,744	34.27	3,218(85.95%)	526(14.05%)
**Use of internet**				
No	10,358	94.78	9,462(91.35%)	896(8.65%)
Yes	570	5.22	417(73.21%)	153(26.79%)
**Contraceptive use**				
Not use	7,762	71.03	7,055(90.89%)	707(9.11%)
Use	3,165	28.97	2,824(89.22%)	341(10.78%)
**Cigarette smoking**				
No	10,865	99.42	9,822(90.40%)	1,043(9.60%)
Yes	63	0.58	57(91.37%)	6(8.63%)
**Alcohol use**				
No	6,896	63.11	6,270(90.91%)	626(9.09%)
Yes	4,031	36.89	3,609(89.53%)	422(10.47%)

In this study, the overall prevalence of overweight/obesity in Ethiopia was 9.6% (95% CI: 9.05–10.1). There were regional variations, with in Addis Ababa, Dire Dawa, Harari and Somalia regions having a higher prevalence of overweight/obesity, while the lowest prevalence of overweight/obesity was found in SNNP and Amhara region of Ethiopia.

### Spatial analysis result

#### Spatial autocorrelation (Global Moran’s I) analysis

**Fig 2:**
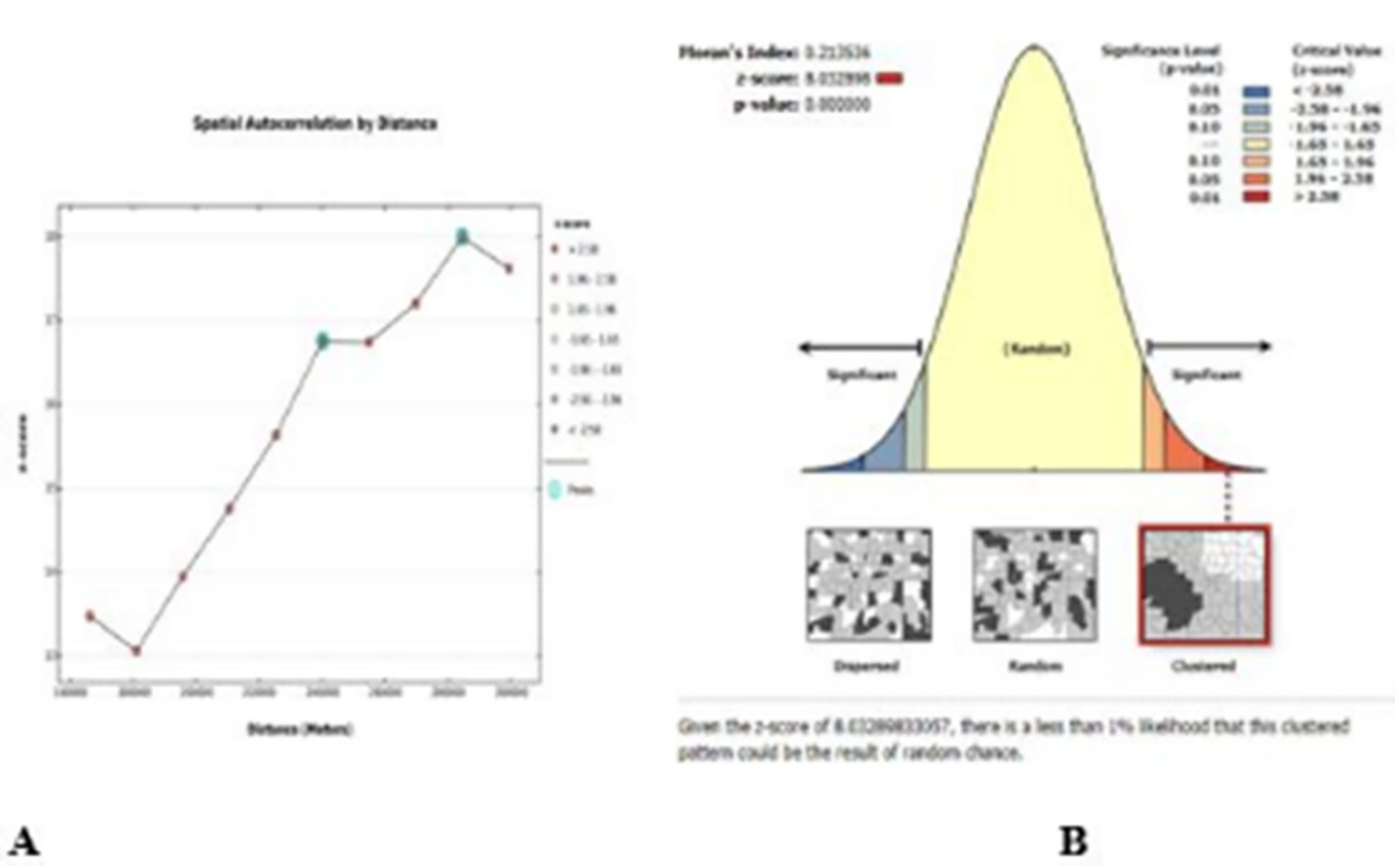
Incremental spatial autocorrelation (A) and spatial autocorrelation (B) of overweight/obesity among reproductive age group women in Ethiopia, EDHS 2016.

The global spatial statistics were determined using Moran’s I value. As shown in the Fig 2 left side, at 284,602 meter distances, statistically significant z scores revealed that spatial factors encouraging clustering are most evident. With a starting distance of 166,201 meters, incremental spatial autocorrelation shows that ten distance bands were detected. As shown in Fig 2 right side, the spatial variation of overweight/obesity among reproductive-age women in Ethiopia was clustered with a Global Moran’s I of 0.21 (p value 0.0001) and a Z-score of 8.03, indicating that there is less than 1% possibility that this clustered pattern is the result of random chance.

#### Getis-Ord Gi* hot spot and cold spot analysis

**Fig 3:**
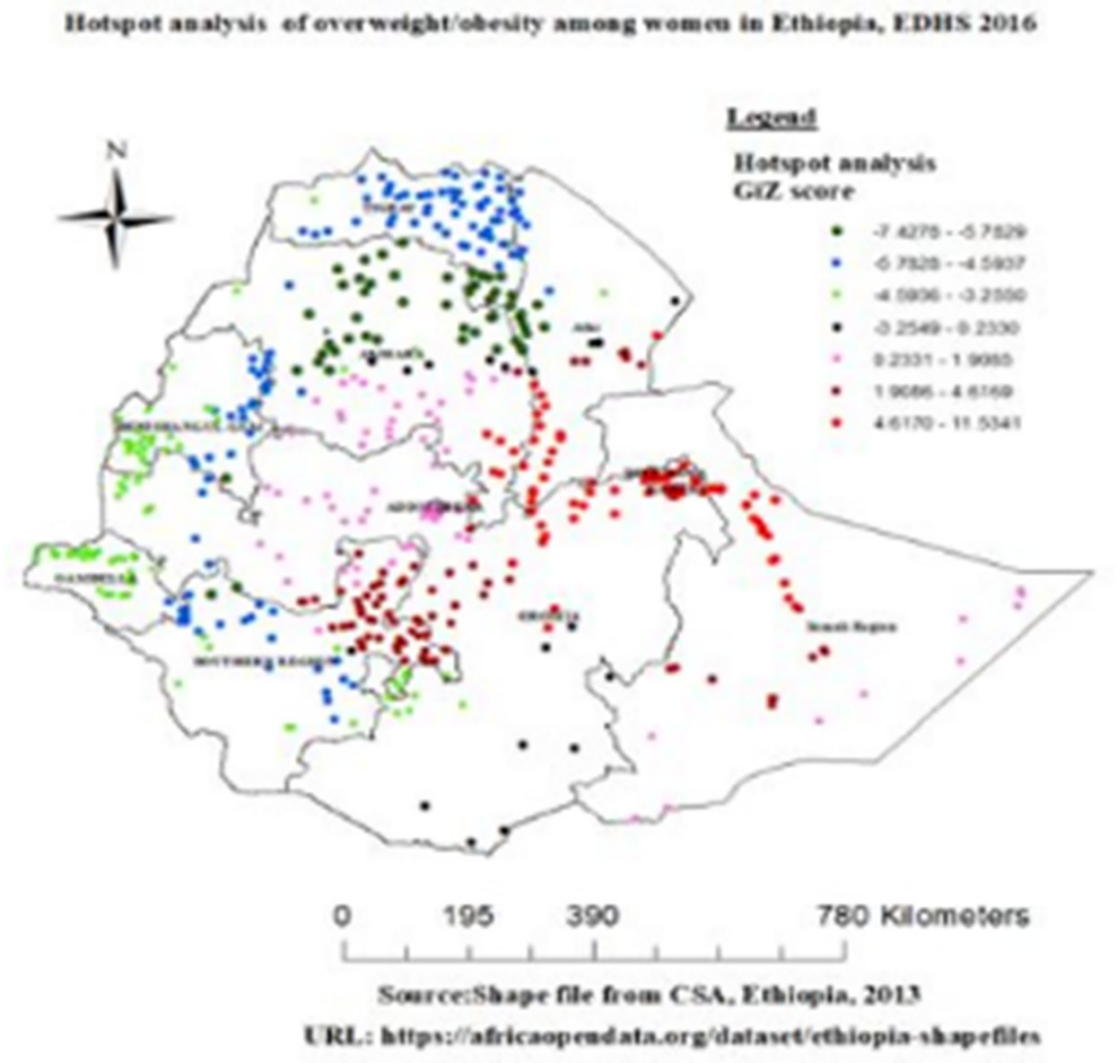
Hot spot analysis of overweight/obesity among reproductive-age women in Ethiopia, EDHS 2016.

As shown Fig 3 below, the spatial analysis at the cluster level shows that statistically significant high hotspots of overweight/obesity were found in Addis Ababa, harrari, Dire Dawa, some part of amhara and afar region, most part of oromia and Somalia region and South Nation Nationality and People region (SNNP); whereas, statistically significant low hotspots of overweight/obesity were found in most parts of Amhara, Tigray, Gambella and Benishangul Gumize region of Ethiopia.

#### Spatial interpolation

**Fig 4:**
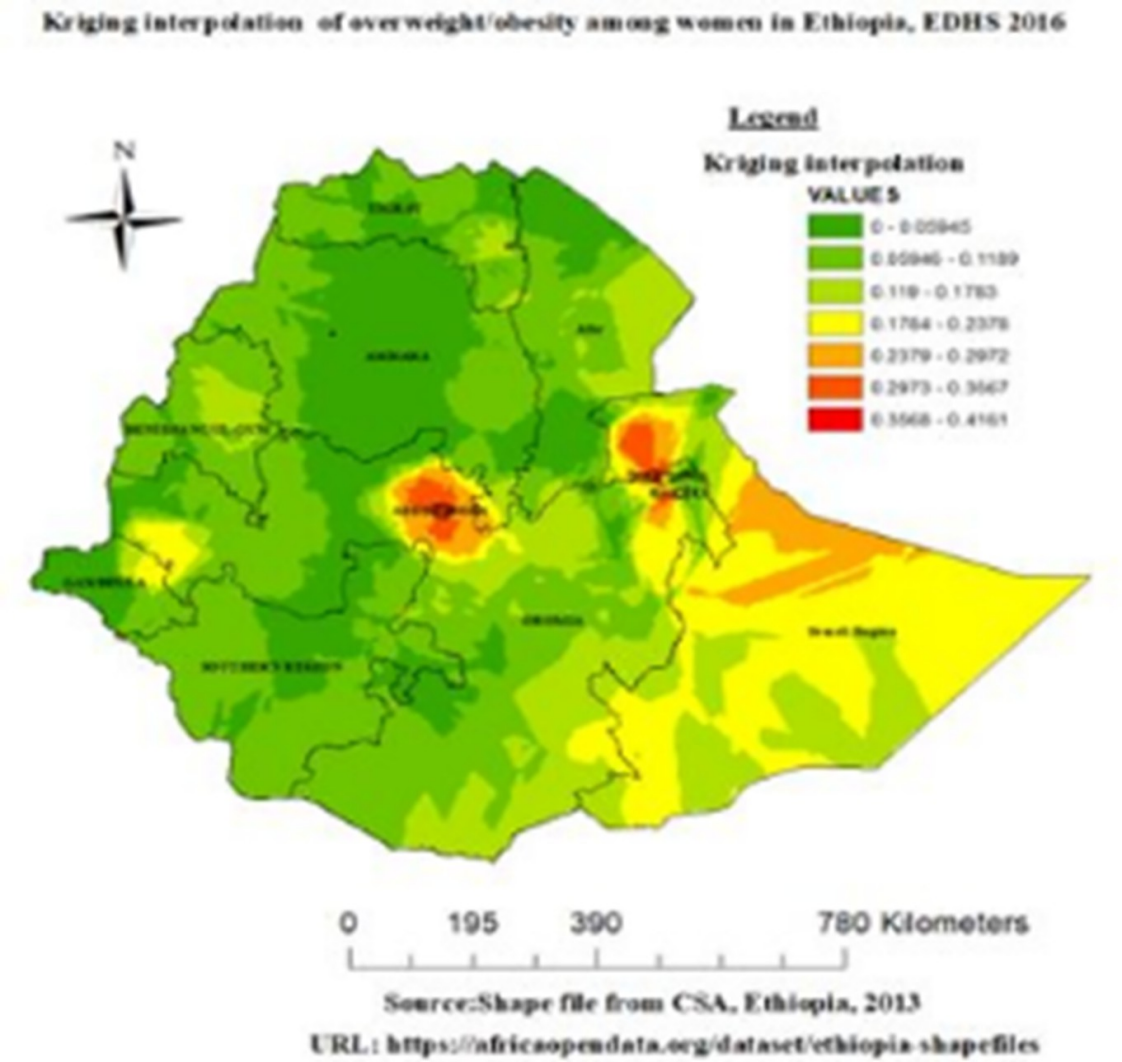
Interpolation of overweight/obesity among reproductive-age women in Ethiopia, EDHS 2016.

The ordinary Kriging spatial interpolation method was used in this study for predicting overweight/obesity in unobserved areas. For interpolation method comparison, the mean and Root-Mean-Square-Error were used. Table 2 proved that, the ordinary Kriging spatial interpolation was the best fit interpolation method with the lowest mean predicted error (MPE: 0.00002) and Root Mean Square predicted Error (RMSP: 0.01092) compared to other interpolation method. Based on ordinary Kriging analysis, in EDHS 2016 the predicted overweight/obesity increases from green to red-colored areas. The red color indicates high-risk areas of predicted overweight/obesity, and the green color indicates low-risk area of predicted overweight/obesity. Fig 4 depict that Addis Ababa, harrari, dire dawa, oromia and Somalia region were predicted as more risky area for overweight/obesity than other regions.

**Table 2:** Interpolation mothed comparison for overweight/obesity, EDHS 2016.

	Parameters	
Interpolation method	Mean error (ME)	Root-mean-square error(RMSE)
**Deterministic interpolation method**		
Inverse distance weighted	0.01368	0.11769
**geostatistical interpolation methods**		
**Ordinary kriging**	**0.00002**	**0.01092**
Simple kriging	0.00098	0.11135
Universal kriging	0.00011	0.01111
Disjunctive kriging	0.00181	0.11162
Probability kriging	0.00613	0.42413
Indictor kriging	0.00620	0.45451

#### Spatial scan statistics

**Fig 5:**
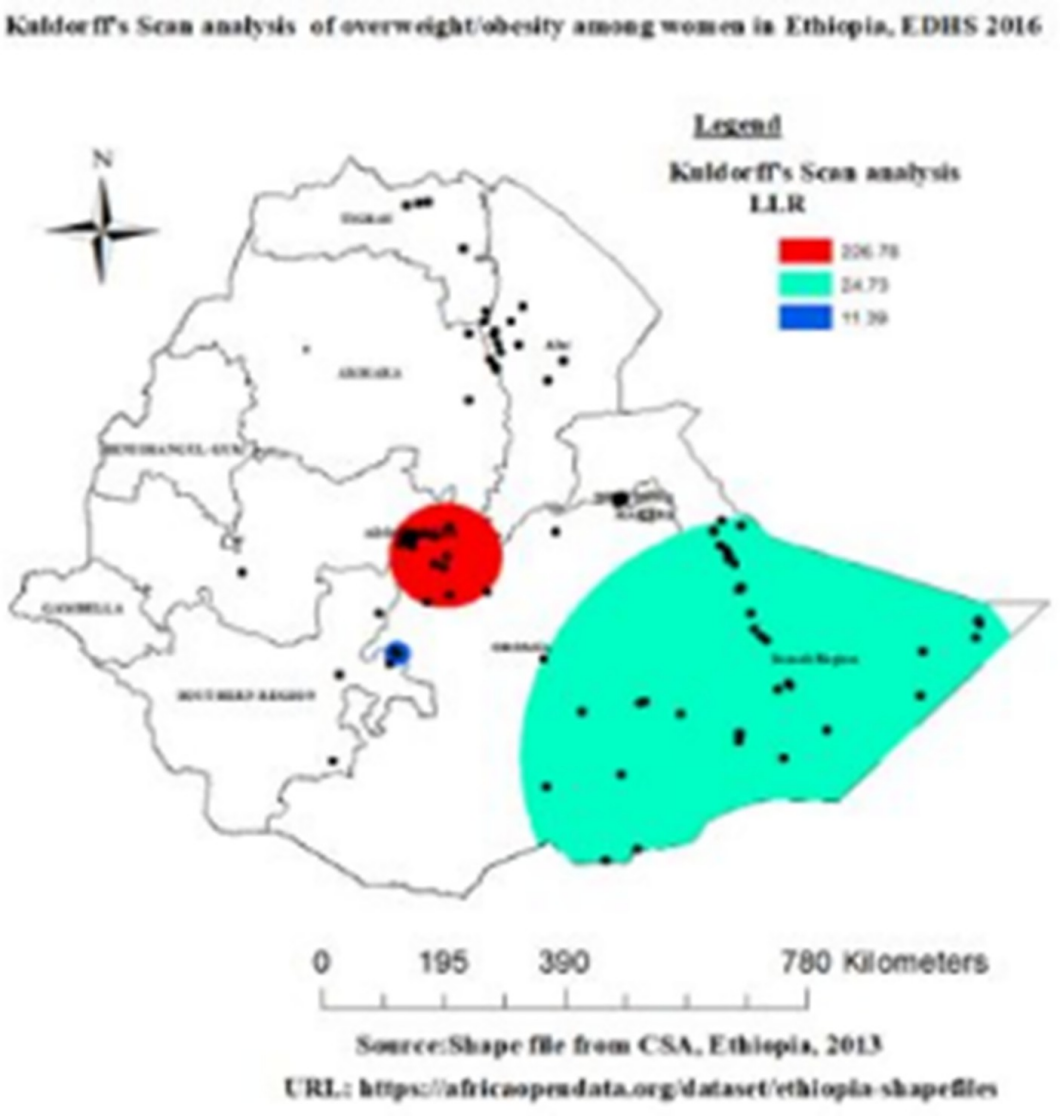
Spatial SaTScan analysis of overweight/obesity among reproductive age group women across regions of Ethiopia, EDHS 2016.

The result from spatial Kuldorff’s Scan analysis, we identified 13 spatial clusters, from which six of them were statistically significant at P-value < 0.05. The primary cluster, the red color ring spatial window was typically located at the central part of the country which encompasses in Addis Ababa, southeast amhara and some part of oromia region. This spatial window was centered at 8.712307 N, 39.296911 E with 90.34 km radius and Log-Likelihood ratio (LLR) of 226.78 with relative risk (RR: 4.17), at p < 0.001. It showed that women within the spatial window had 4.17 times higher risk of overweight/obesity than women outside the window (Fig 5 and Table 3).

**Table 3:** Significant clusters of overweight/obesity among reproductive age women, EDHS 2016.

Type of cluster	# cluster location	#pop	# case	RR	LLR	Coordinate/radius	p-value
Primary cluster	66	1232	358	4.17	226.78	8.712307 N, 39.296911 E/90.34 Km	< 0.001
Secondary cluster 1	41	204	54	2.88	24.73	5.589269 N, 44.175032 E/420.35 km	< 0.001
Secondary cluster 2	3	131	31	2.53	11.39	7.193591 N, 38.593712 E/21.17km	< 0.001
Secondary cluster 3	1	145	64	4.87	60.45	8.453819 N, 36.343666 E/0 km	< 0.001
Secondary cluster 4	1	58	36	6.71	48.84	9.090824 N, 40.873894 E/0 km	< 0.001
Secondary cluster 5	1	50	19	4.04	14.67	6.876749 N, 37.757759 E/0 km	< 0.05

N:B LLR: Log-Likelihood ratio, RR: Relative Risk

In addition, the remaining two spatial windows with tourmaline green and blue colors were secondary clusters. The tourmaline green color spatial window covers eastern parts of oromia and Somalia region this spatial window was centered at 5.589269 N, 44.175032 E with 420.35 km radius and Log-Likelihood ratio (LLR) of 24.73 relative risk (RR: 2.88), at p < 0.001. It showed that women with in the spatial window had 2.88 times higher risk of overweight/obesity than women outside the window. Whereas, the blue color spatial window covers northeast parts of SNNP and western parts of oromia region this spatial window was centered at 7.193591 N, 38.593712 E with 21.17 km radius and Log-Likelihood ratio (LLR) of 11.39 relative risk (RR: 2.53), at p < 0.005. It showed that women with in the spatial window had 2.53 times higher risk of overweight/obesity than women outside the window. However, the fourth, fifth and the sixth spatial windows of the detected secondary cluster were not plotted, because the clusters were detected only a single location identification (ID) (Fig 5 and Table 3).

#### Spatial regression analysis

*Factor affecting spatial variation of overweight/obesity*. ***Ordinary Least Square (OLS) model result*.** Table 4 illustrates the results from an OLS model of overweight/obesity. The OLS model explained about 55.8% (Adjusted R square = 0.558) of the variation in overweight/obesity and all of the OLS method’s assumption were met. The coefficients represent the strength and the type of each explanatory variable and the overweight/obesity. We used the robust probability to determine the statistical significance of the coefficients because the Koenker (BP) statistic was significant and all the coefficients were statistically significant (p < 0.01). The Joint Wald statistic was also statistically significant (p< 0.01), indicating that the total model was significant. There is no multicollinearity between explanatory variables (Variance inflation factor (VIF) < 7.5), according to (Table 4). In addition, the Jarque-Bera statistic was non-significant indicating the model residuals were normally distributed. Since Koenker test was statistically significant (Koenker (BP) Statistics = 17.99 p-value < 0.001), it indicates relationships between some or all of explanatory variables and dependent variable is non–stationary which reveals the difference of coefficients across enumeration areas. In the context of this finding the Koenker (BP) test proved significant, enabling for the execution of geographically weighted regression.

**Table 4:** Global beta coefficients of the ordinary least square model summary and diagnostics for overweight/obesity among reproductive-age women in Ethiopia, EDHS 2016.

Variable	Coefficient	Std. error	Probability	Robust probability	VIF
Intercept	-0.042753	0.015853	0.007190*	0.005172*	-----
Maternal age 35–39	0.110525	0.044224	0.012696*	0.022029*	1.0447
Maternal age 40–44	0.114981	0.050372	0.022778*	0.016092*	1.0286
Rich	0.113904	0.019679	0.000006*	0.000014*	3.8055
Not working	0.164364	0.017489	0.000266*	0.000139*	1.1671
Watching Tv	0.173382	0.025442	0.000000*	0.000000*	5.1663
Internet Use	0.238863	0.052486	0.000009*	0.000200*	2.4518
OLS diagnostics					
Diagnostic criteria		Magnitude		p-value	
AICc		-1063.21			
R squared		0.562			
Adjusted R squared		0.558			
Joint F-Statistics		131.69		0.00000*	
Joint Wald Statistics		791.43		0.00000*	
Koenker (BP) Statistics		17.99		0.006243*	
Jarque-Bera Statistics		109.90		0.093714	

NB**: AICc**: Akaike’s Information Criterion

#### Geographical weighted regression (GWR) analysis

The OLS regression identified predictors of overweight/obesity. However, it is a global model that assumes the relationship between each explanatory variable and overweight/obesity were stationary across the study area. Table 5 depicts that GWR model for overweight/obesity in the study area.

**Table 5:** Geographic weighted regression (GWR) model for overweight/obesity in Ethiopia, EDHS 2016.

**Explanatory variable**	Women age (35–39 and 40–44 years), women with rich wealth index, women who had watching TV and internet use and women who had not currently working
**Residual square**	4.23
**Effective number**	81.49
**Sigma**	0.08
**AICc**	-1198.02
**Multiple R square**	0.710
**Adjusted R square**	0.667

NB**: AICc**: Akaike’s Information Criterion

In the geographically weighted regression model, selected predictor variables was fitted. Both the Ordinary Least Square (OLS) and Geographical Weighted Regression (GWR) data was fitted for model compression. For model comparison, the corrected Akakie Information Criteria (AICc) and adjusted R2 were used. Table 6 proved that, Geographical Weighted Regression was the best fit model with AICc of -1198.02 compared with -1063.21 as well, the GWR model best explained by the predictor variables for overweight/obesity among reproductive age group women with an adjusted R2 value 66.7% compared to OLS adjusted R2 value 55.8%.

**Table 6:** The Model comparison between the OLS model with the GWR model.

Variable	OLS	GWR
AICc	-1063.21	-1198.02
Adjusted R squared	0.558	0.667

**NB: AICc =** Akakie Information Criteria**, OLS:** Ordinary Least Square**, GWR:** Geographically Weighted Regression

**Fig 6:**
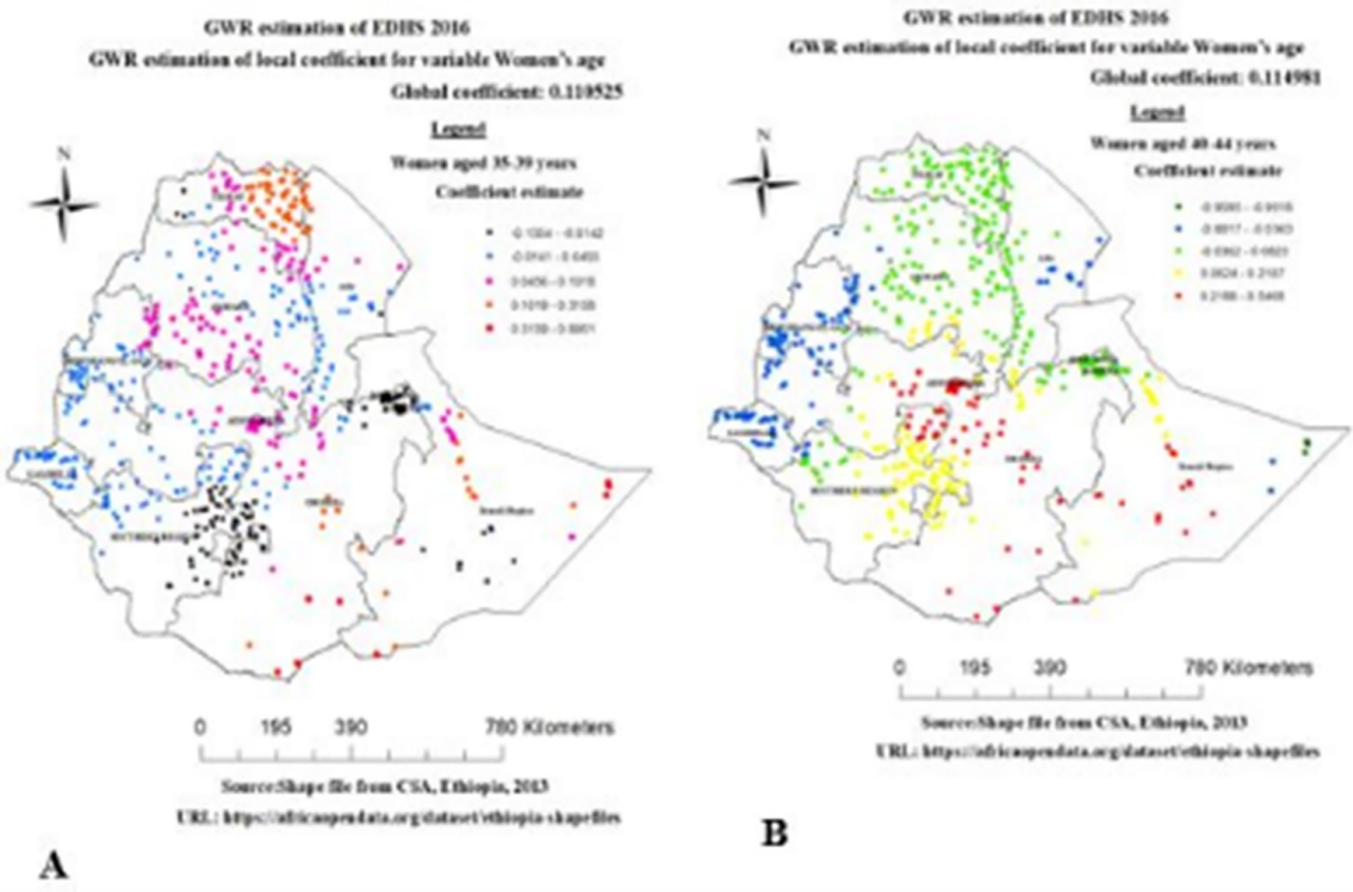
Geographically varying values of coefficients per cluster for predictor women aged 35–39 years (A) and 40–44 years (B), EDHS 2016.

In Geographically weighted Regression model predictor variables women from rich household wealth status, women’s age group between (35–39 and 40–44 years), women`s who do not currently working, women with watching Tv and internet use were statistically significant predictors spatially for overweight/obesity among reproductive age women in Ethiopia. Women age had different statistical significance in different parts of Ethiopia for overweight/obesity among reproductive-age women. The coefficients of women aged (35–39 years) spatially vary from -0.1354 to 0.8951, which indicates both negative and positive effect on overweight among reproductive age group women in Ethiopia. As shown in the Fig 6 left side, women aged (35–39 years) had a strong and positive relationship with overweight/obesity were observed in Tigray, Addis Ababa, southern parts of Amhara, some part of oromia and Somalia regions. Similarly, the coefficients of women aged (40–44 years) spatially vary from -0.9595 to 0.5468, which indicates that both a negative and positive effect spatially on overweight/obesity. The coefficients of women aged (40–44 years) had a strong and positive relationship with overweight/obesity in Addis Ababa, SNNP, southern parts of Amhara, oromia and Somalia regions (Fig 6B).

**Fig 7:**
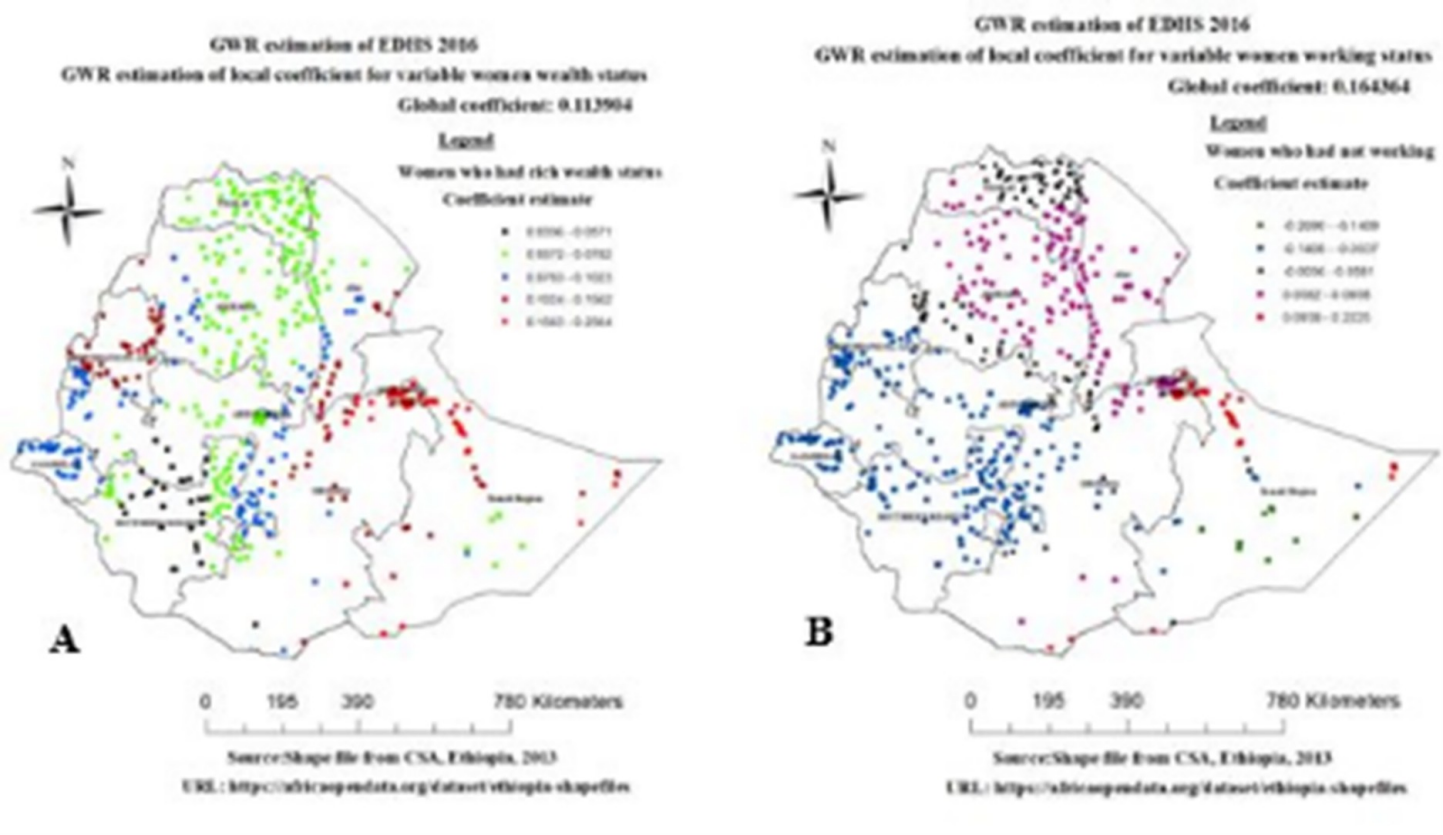
Geographically varying values of coefficients per cluster for predictor rich wealth index (A) and not working (B), EDHS 2016.

Rich wealth status of the household had different statistical significance in different parts of Ethiopia for overweight/obesity among reproductive age group women. The coefficients of rich wealth status spatially vary from 0.0336 to 0.2564, indicate that the effect of association different across regions of Ethiopia. Rich wealth status was statically significant in overweight/obesity in oromia, southern and eastern parts of afar, harrari, some part of Somalia, Benishangul gumize, Gambella some part of SNNP, Addis Ababa and Dire Dawa administrative city (Fig 7A). Women who had not currently working status was statically significant for overweight/obesity over the region of Ethiopia. The coefficients of not currently working vary spatially from -0.2690 to 0.2225, which indicates women who had not currently working status had a negative and positive effect spatially for overweight/obesity. Not currently working status was statically significant and positive associated with overweight/obesity in amhara, afar, harrari, Dire Dawa, Tigray and northern part of oromia and Somalia region (Fig 7B).

**Fig 8:**
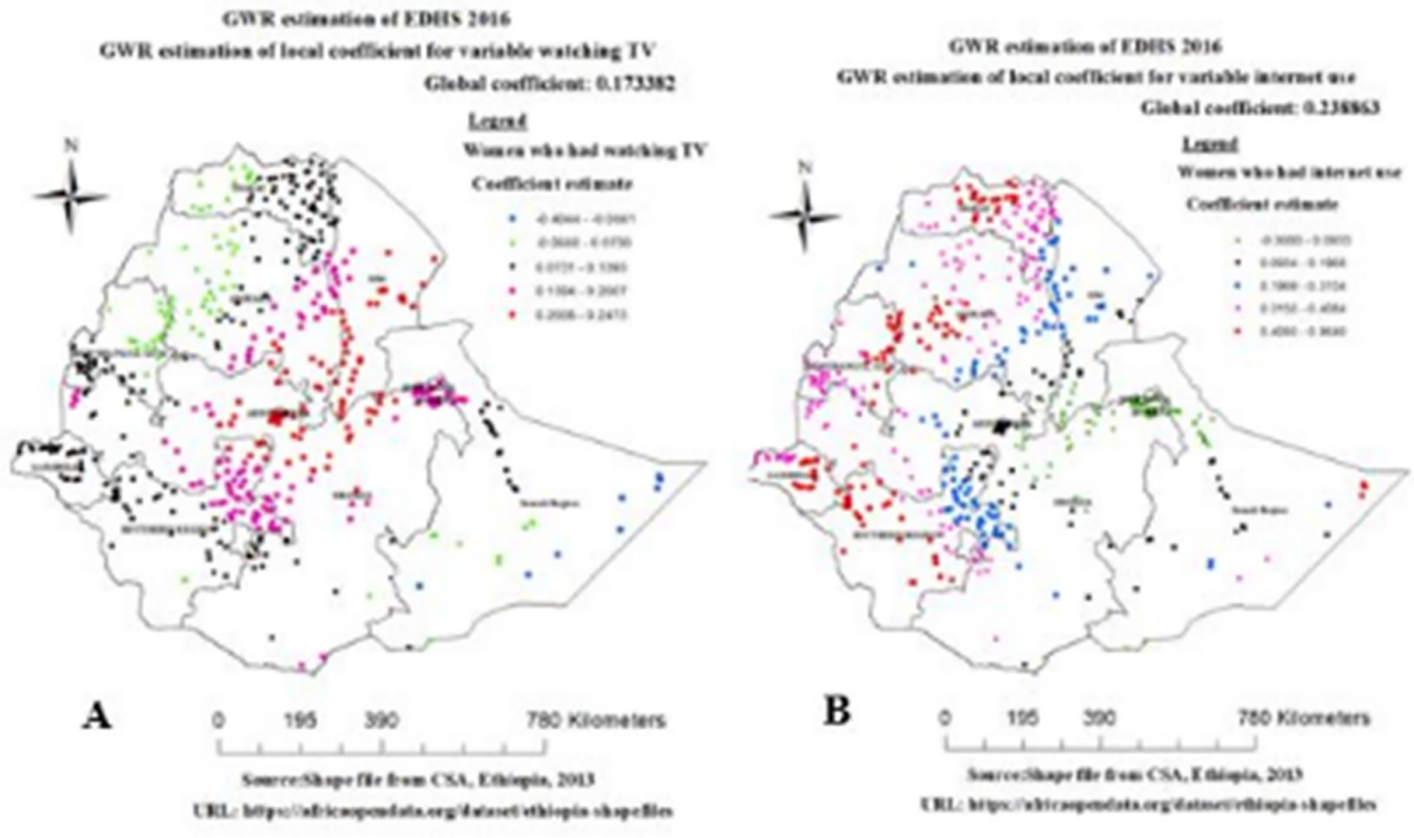
Geographically varying values of coefficients per cluster for predictor watching Tv (A) and internet use (B), EDHS 2016.

In addition, frequency of watching TV was statistically significant different part of Ethiopia. The coefficients of watching TV vary spatially from -0.4044 to 0.2473, which indicates women with watching TV had a negative and positive effect spatially for overweight/obesity. Watching TV had a strong and positive relationship with overweight/obesity were observed in Addis Ababa, Dire Dawa, harrari, Amhara, and most parts of Afar, SNNP and oromia regions (Fig 8A). Internet use was statistically significant different part of Ethiopia. The coefficients of internet use vary spatially from -0.0080 to 0.9640, which indicates women with internet use had a negative and positive effect spatially for overweight/obesity. Internet use had a strong and positive relationship with overweight/obesity in Addis Ababa, Tigray, amhara, Gambella, eastern part of Somalia, SNNP region of Ethiopia (Fig 8B).

## Discussion

This study aimed to determine the spatial variations of overweight/obesity and its associated factors among reproductive age group in Ethiopia using geospatial statistical models. Our spatial analysis showed the presence of spatial heterogeneity using a significant positive spatial autocorrelation identified by Moran’s I statistic as well as the significant local clusters identified by Getis-Ord Gi* analysis across the region. The spatial distribution of overweight/obesity among reproductive-age women in Ethiopia was non-random, with higher levels of correlation in overweight/obesity rates between Ethiopian regions. Some plausible factors for neighborhood features could explain overweight/obesity spatial patterns. The residents may share similar lifestyles about diet, nutritional habits and physical activity, it’s also possible that residents of nearby places with similar obesity rates share activities that contribute to the condition [23].

The Getis-Ord Gi* analysis revealed that a Significant overweight/obesity highly clustered at Addis Ababa, harrari, Dire Dawa, some part of amhara and afar, most part of oromia and Somalia and South Nation Nationality and People region. Spatial Kuldorff’s Scan statistics analysis revealed that a total of 66 statistically significant primary clusters were identified. The significant primary clusters were observed in Addis Ababa, southeast amhara and some part of oromia region. Respective literature also revealed that the presence of geographical variation for overweight/obesity. This finding is supported by studies done in Ghana, a Significant clustering (Moran’s I = 0.3145 (p<0.05)) of women with high BMI values were observed in the Greater Accra, Central, Western and Ashanti regions [10]. Similarly, a study done in Uganda, a significant cluster were observed in Kampala, Mbarara, Masaka and a smaller city, Kabal [24]. Furthermore, this finding supported by the study done in Canada a significant cluster and high hot spot values were detected in Atlanta, northern health region of Alberta, Saskatchewan, Manitoba and Ontario [25] This might be rapid urbanization and modernization to the escalating overweight prevalence in developing nations [26]. In addition, this might be due to the sociocultural and socioeconomic differences between women in different regions [27].

The spatial regression analysis revealed that statistically significant and a positive relationship predictor variables of overweight/obesity were identified. women’s from rich wealth status household, women aged (35–39 years) and (40–44 years), women`s who had not currently working, women with watching Tv and internet use were significant predictors spatially for overweight/obesity among reproductive age women in Ethiopia. The GWR coefficients of the strong predictors of overweight/obesity across Ethiopia, for instance, range from 0.0336 to 0.2564 for women with rich wealth index household.

Different studies conducted in both developed and developing countries revealed the existence of considerable significant difference of overweight/obesity predictors across a geographical area among reproductive age group women [10, 25, 28]. The findings of this study reveal a strong and positive link between women with rich wealth index and overweight/obesity. This finding is agreed with different studies done elsewhere [29–31]. Similarly, the finding of this analysis stated that a household with a rich wealth quintile was a strong and positive predictor of overweight/obesity in oromia, southern and eastern parts of afar, harrari, some part of Somalia, Benishangul gumize, Gambella, some part of SNNP, Addis Ababa and dire dawa administrative city. Literature show that the highest proportion of women with rich wealth index observed in Addis Ababa (99.9%), dire dawa (60.7%), harrari (54.4%) and Gambella (34.4%) [15]. This could be wealthy women can afford to buy a wide variety of foods and have easy access to sugary, energy-dense commercialized products, leading to over nutrition relatively, women with poor economic status have a low opportunity of affording varieties of nutritious diet [32]. Furthermore, women in higher wealth quintiles have a tendency to live sedentary lifestyles and consume more energy-dense foods.

The women age groups (35–39 and 40–44 years) had a favorable and substantial relationship with overweight/obesity in this study. This finding is supported by studies from different countries such as in Bangladesh [11], Kenya [33], Nigeria [34], Ghana [10] and regional and nation level in Ethiopia [8, 35]. Furthermore, the current study found a robust and strong relationship between women’s age (35–49 and 40–44 years) and overweight/obesity in Tigray, Addis Ababa, and southern amhara, some parts of Oromia, SNNP and Somalia. The possible reason for this finding may be that old age is likely to be characterized by high physical inactivity as well as the consumption of more energy-dense food, which may result in overweight/obesity [36, 37]. Furthermore, as age increases, people tend to have a sedentary lifestyle contributing to an increase in body mass composition [38].

In this study, women who were not currently worked had a positive and significant relationship with being overweight/obesity. This conclusion is corroborated up by evidence of other investigations [39, 40]. Moreover, the current study identified a positive relationship between women who had not currently working and overweight/obesity in amhara, afar, harrari, dire dawa, tigray and northern part of oromia and Somalia region. Evidence suggest that high proportion of unemployment rate was found in afar (30.1%), somalia (28.7%), dire dawa(27.2%), harrari(17.9%) and amhara(12.8%) [41]. The possible reason may be women those who had not working is more likely to be characterized by insufficient physical activity, insufficient physical activity was more strongly associated with prolonged sedentary behavior with overweight/obesity [42]. Another possible reason not working association with watching of television and frequency of internet use. Evidence has shown that substantial connections between TV watching and obesity were discovered in non-workers but not in workers, showing that light-intensity and intermittent activities during work are protective against overweight/obesity [43].

We included variables of mass media exposure to women in this study, because the media is a beneficial and widely available source of information for disseminating health information on proper diet, good behavior, and a healthy lifestyle. This study discovered that women who watch television and use the internet have a strong and positive correlation to being overweight/obesity. This finding was in agreement with the study done in India [44], Ghana [45], Nepal [46] and Ethiopia [8, 47]. Moreover, This study show that women who watched television and utilized the internet had a high and positive link to having overweight/obesity in Addis Ababa, Dire Dawa, harrari, Amhara, and some parts of Afar, SNNP, oromia regions, Tigray, Gambella and eastern part of Somalia. Literatures suggested that the highest proportion of women used internet were in Addis Ababa (32.9%), in harrari (12.6%), in Gambella (6.6%) and Dire Dawa (18.7%). Similarly, the highest proportion of women who watch TV were 80.8%, 33.2% and 47.5%, 40.5% and in Addis Ababa, harrari, Dire Dawa and Gambella respectively [15]. The possible reason may be women those who had watching television and internet use more likely to be reducing physical activity. Similarly, advertisements selling calorie-dense, unhealthy meals are more effective and likely to reach people who are watching television for longer periods of time and combined with an already sedentary lifestyle, increases their chances of becoming overweight/obesity [48].

### Strength and limitation

The utilization of nationally representative data with a large sample was the study’s key strength. Furthermore, this study had used geographically weighted regression analysis that could enables to determine local coefficients a step advance from ordinary least square analysis. This study also its own limitations such as, food availability, intake, Physical activity level and other dietary habits of women are not included in the EDHS data, which could have an impact on their BMI. In addition, the EDHS data, like other cross-sectional data, cannot be used to establish a causal association. Furthermore, we removed 21 clusters from the analysis because they lacked coordinated data and had missing data, which may have influenced the estimated result. Therefore, the interpretation or conclusion based on this study should consider these limitations.

## Conclusion

In Ethiopia, overweight/obesity varies across the region. Statistically, significant-high hot spots of overweight/obesity were detected in Addis Ababa, Harari, Dire Dawa, some parts of Amhara and afar region, most of the Oromia and Somalia region, and the South Nation Nationality and People region of Ethiopia. In spatial regression analysis, women with ages between 35–39 and 40–44 years, women who had watched TV and internet use, women with a rich wealth index, and women who had not currently working were statistically significant predictors (at a local level) in different regions of Ethiopia. Therefore, the ministry of health and the Ethiopian public health institute, try to initiate policies and practices that could include providing funding for physical education as well as recreational centers in communities most in need. In addition, public and private mass media create awareness of healthy lifestyles is promoted by health education regarding increased physical activity and reduced sedentary behavior through various media platforms. Moreover, researchers to have a research on data that incorporate food availability, consumption, and other dietary habits of women.

## Abbreviations

AICc: Akakie Information Criteria
BMI: Body Mass Index
CI: Confidence Interval
CMHS: College of Medicine and Health Sciences
CSA: Central Statistics Agency
DHS: Demographic and Health Survey
EAs: Enumeration Areas
EDHS: Ethiopia Demographic and Health Survey
GWR: Geographically Weighted Regression
LLR: Log-Likelihood ratio
NCDs: Non-Communicable Diseases
OLS: Ordinary Least Square regression
RRs: Relative Risks
SDG: Sustainable Development Goals
SNNP: Southern Nations, Nationalities, and People’s
TV: Television and WHO: World Health Organization
WHO: World Health Organization
